# Analysis of a Pulse Rate Variability Measurement Using a Smartphone Camera

**DOI:** 10.1155/2018/4038034

**Published:** 2018-02-05

**Authors:** András Bánhalmi, János Borbás, Márta Fidrich, Vilmos Bilicki, Zoltán Gingl, László Rudas

**Affiliations:** ^1^Institute of Informatics, University of Szeged, Szeged, 2 Árpád Square 6720, Hungary; ^2^2nd Department of Internal Medicine and Cardiology Clinic, University of Szeged, Szeged, 6 Semmelweis Street 6725, Hungary; ^3^Department of Anesthesiology and Intensive Therapy, University of Szeged, Szeged, 6 Semmelweis Street 6725, Hungary

## Abstract

**Background:**

Heart rate variability (HRV) provides information about the activity of the autonomic nervous system. Because of the small amount of data collected, the importance of HRV has not yet been proven in clinical practice. To collect population-level data, smartphone applications leveraging photoplethysmography (PPG) and some medical knowledge could provide the means for it.

**Objective:**

To assess the capabilities of our smartphone application, we compared PPG (pulse rate variability (PRV)) with ECG (HRV). To have a baseline, we also compared the differences among ECG channels.

**Method:**

We took fifty parallel measurements using iPhone 6 at a 240 Hz sampling frequency and Cardiax PC-ECG devices. The correspondence between the PRV and HRV indices was investigated using correlation, linear regression, and Bland-Altman analysis.

**Results:**

High PPG accuracy: the deviation of PPG-ECG is comparable to that of ECG channels. Mean deviation between PPG-ECG and two ECG channels: RR: 0.01 ms–0.06 ms, SDNN: 0.78 ms–0.46 ms, RMSSD: 1.79 ms–1.21 ms, and pNN50: 2.43%–1.63%.

**Conclusions:**

Our iPhone application yielded good results on PPG-based PRV indices compared to ECG-based HRV indices and to differences among ECG channels. We plan to extend our results on the PPG-ECG correspondence with a deeper analysis of the different ECG channels.

## 1. Introduction

Heart rate variability (HRV) is a rarely used clinical term, but it provides useful information about the variation between consecutive heart beats. HRV parameters could help to describe the activity of the autonomic nervous system (ANS), and through this, we can get a better picture about the status of our health [1].

Most of previous studies in the area of HRV measurements just focused on the technical or the medical aspects. Studies describing relations between these two are quite rare. Many of the medical studies do not investigate the new analytical methods, and most of the new methods have not been validated in medical experiments. The value of our study is based on this economically and medically relevant problem, and we try to solve it using tools taken from information technologies [2]. A medically relevant problem is, for example, the diagnosis and treatment of the cardiovascular diseases, which are the cause of the 37% of global mortality (2012), corresponding to as many as 17.5 million people. Out of these, 6 million people were under 70 years old and 7.4 million of them died from coronary artery disease [3]. The relation between HRV and coronary artery diseases was found relatively early [1]: “The observation that in patients with an acute MI, the absence of respiratory sinus arrhythmias is associated with an increase in “in-hospital” mortality represents the first of a large number of reports which have demonstrated the prognostic value of assessing HRV to identify high-risk patients.”

Hence, this is why it would be beneficial to build up a widely accessible service, which can be used easily to measure HRV not just in a hospital but anywhere. This service could improve the quality of life and survival chances of those who are diagnosed with such problems. However, there is a lack of systematic statistical evaluation of the additive prognostic value of the new methods [2]. Over the past 30 years, the literature has not provided much support for the real clinical utilization of HRV. It is anticipated that new methods will aid studies involving large populations, which hopefully will allow us to expand our physiological knowledge and improve our understanding of its clinical relevance [2]. Previously published articles presented new methods and analytical techniques on this topic [4–8]. However, a breakthrough has not been achieved since we have so far failed to collect a critical amount of data from either healthy or sick populations [2].

**Figure 1:**
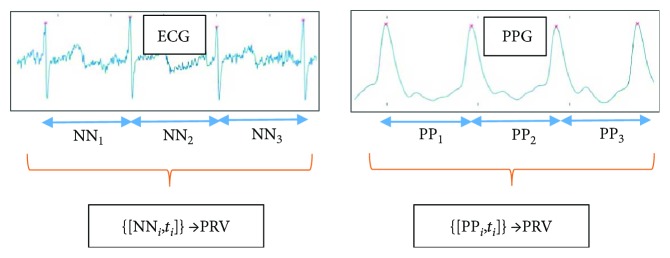
Connection between HRV and PRV analysis. From the ECG signal, the NN intervals (time durations) are determined, with the corresponding timestamps. The timestamps are needed when spectral analysis is applied to the NN time serial. The data for PRV is similarly obtained from the PP durations between the consecutive maximum values in the PPG signal.

A new HRV registration technique is needed that can record professionally validated data even by a layman, anytime and anywhere. One possible method might be photoplethysmography (PPG), which can be used to measure pulse rate variability (PRV) on the limbs. PRV and HRV have good correlations, and it was confirmed by several previous publications [9–11]. The relation among the HRV, ECG (electrocardiogram), PRV (pulse rate variability), and PPG (photoplethysmogram) is shown in Figure 1. ECG is a voltage signal, while PPG is the time serial got from digitalizing the measurements of the reflected or absorbed light, which changes with the periodic blood flow. PRV can be easily measured with the help of a smartphone flash and camera [12, 13] (or using other low-cost tools [14]). The operation of PPG is well formulated by the authors of [15]: “PPG is measured via reflection through the illumination of the skin using an LED (e.g., the smartphone's flash) and through the detection of the amount of light that is reflected by a photodetector or a camera located next to the light source. The resulting PPG signal is composed of a direct current (DC) component, which varies slowly depending on tissue properties and blood volume. The alternating current (AC) component varies more rapidly to detect the pulsatile factor. After cardiac systole, local blood volume increases acutely, reducing the received light intensity. During diastole, blood volume decreases and light reflection increases.” The intuitive explanation behind the theory of substituting heart rate with pulse rate lies in the common physiological origin of the two signals. However, the ECG signal is an electrical voltage signal, and the PPG signal is measured by light reflection or absorption; the maximum values of both signals are related to cardiac systole.

Using a smartphone as a PPG makes the registration more user friendly than previous ECG measurement techniques. It should be mentioned here that there are some solutions for measuring ECG using smartphones [16–18], and these solutions involve additional devices connected to a smartphone via a cable or radio connection. Although these techniques are easy to use, these still require third-party devices and extra cost. When PPG is measured with a smartphone, no external devices and expensive accessories are required. Nowadays, smartphones are widespread and they can be used for various telemonitoring purposes [19]. In this way, we give patients the chance to control their own monitoring and health. The evaluation can be carried out instantly by using new algorithms run by healthcare professionals, which can be accessed from anywhere via the Wi-Fi or 3/4G Internet. During our study, we examined the acceptability of using “stand-alone” smartphone-based PRV registration with a PPG technique in clinical settings instead of the complicated ECG-HRV registration. We created smartphone-based software to measure PRV with high quality. Then, an environment was devised to measure PPG and ECG at the same time for the sake of an accurate validation. Our aim was to develop a PRV measurement technique, which is widely available and can replace the ECG-based HRV measurements.

This paper is organized as follows. In Section 2, we give a detailed description of how the parallel ECG and PPG measurements were taken. In the same section, the indices derived from the ECG and PPG signals are defined, and a commonly used comparison methodology is introduced. In Section 3, we collect our measurement results, and then, we compare the computed PRV and HRV indices using the usual methodology. Afterwards, we introduce an additional validation aspect, which should be taken into consideration in other comparison studies. Essentially, in the previous studies, the PRV was compared with the HRV using just one channel of ECG as the gold standard. However, the HVR indices derived from different ECG channels also show a nonnegligible deviation, and these correspondences among ECG channels are also investigated. The PRV-HRV correspondence is related to the HRV-HRV correspondence. Finally, in Section 4, we draw some pertinent conclusions and make some suggestions for future work.

## 2. Methods and Materials

We will describe the validation methodology, the way that ECG and PPG were recorded in parallel with the intention of having an adequate analysis. Next, we introduce the commonly used HRV parameters, for which the correspondences were investigated in other studies. Then, we describe the comparison methods that are commonly applied to investigate the correspondence between PRV and HRV indices.

The main goal of the study outlined here was to develop a measurement tool that can measure the PRV (pulse rate variability) accurately, and this application can be readily used by a layman. All these requirements can be satisfied using a widely accessible tool called a smartphone like an iPhone 6. There were similar developments in the past, using other types of smartphones [12, 13, 20], but the device and implementation only permitted a low-frequency PPG measurement. The PPG measurement was compared to ECG statistics in that literature, and there was found to be a good correspondence between the HRV (heart rate variability) and PRV parameters. The iPhone 6 smartphone supports a 240-frame-per-second (FPS) video recording, the so-called “Slow-mo” video, and based on this feature, our plan was to develop a PPG measurement application with a sampling rate of 240 Hz. Another goal of this study was to compare our PRV measurements with those HRV parameters computed from the gold standard ECG signals and also to investigate our comparison results among other experiment results like those of [12, 13, 21]. Because different comparison methodologies were used in different research studies, we collected many of the HRV feature computation and comparison methods for the purpose of a thorough investigation.

Later, we investigate a question raised during our comparison process. If there is more than one ECG channel, which channel should be treated as a gold standard? If the ECG device measures just one channel, then, can that measurement be accepted as a gold standard? If there is difference between the statistics calculated from the HRV belonging to different ECG channels, then, the HRV-PRV comparison methodology can also be applied on the different channels of the ECG device. With the results, we should be able to characterize the variability between the channels of the ECG device involved in our investigations. One could compare the PRV-HRV correspondence with the HRV(i)-HRV(j) correspondence; however, up till now, we could not find a similar approach in other studies. So we think that the ECG should be treated as a gold standard including the variance analysis among the derived values got from the different channels.

The measurements were collected from 50 people. Two signals were recorded in parallel from each, namely, an ECG signal (multiple channels) and a PPG signal. The subjects of the experiments were presumably healthy young or middle-aged people (39 males, 11 females; mean age: 27 years). The length of the recordings made was 5 minutes, whose duration is standard in several medical examinations [1, 22]. The participants were asked to sit in a relaxed position and not to speak to others while the measurements were being taken to avoid collecting a lot of artifacts.

### 2.1. Measuring ECG

ECG signals were recorded using a “Cardiax PC-ECG” device. This type of ECG recording device was chosen for several reasons. This device has reusable clamp electrodes, which allow one to record many subjects easily. More importantly, the recorded signal can be easily saved and converted for a further analysis. Many other ECG devices cannot export the recorded data in an appropriate format, the data are stored in a special format, or the data cannot be accessed. The device was connected to the four limbs of the subjects, which allowed us to collect three channels of ECG signals. The sampling frequency of the signals was 500 Hz, and the device filtered the signal with a notch filter (50 Hz), with a high-pass filter (0.01 Hz), and with a low-pass filter (150 Hz).

After collecting the ECG and PPG signals, the same preprocessing steps were performed digitally on all the raw data. Here, we applied a second-order low-pass Butterworth filter with a cut-off frequency of 80 Hz and a second-order high-pass Butterworth filter with a cut-off frequency of 1 Hz. These transformations effectively reduced the noise from high frequencies and slow changes in the signal.

**Figure 2:**
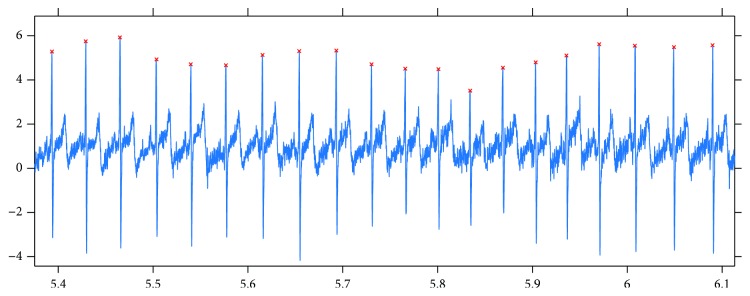
Results got from applying our peak detection method to an ECG signal.

The next step was to find the peaks in the signals. For this purpose, first, a window length was estimated which corresponded to an average RR duration. The estimation was based on finding the first local maximum in the autocorrelation function computed on the signal. Then, with a moving window whose size is slightly larger than that of the estimated average RR interval (e.g., multiplied by 1.3), the maxima were collected in each window, and after filtering out the maxima on the borders, the set of peaks was determined. This method worked well, which is demonstrated by the fact that after a human review of the automatically detected peaks, there were no false or missing peaks found.  Figure 2 shows the results of the peak finding method that we applied here.

Although the participants of the experiments were asked to sit in a relaxed position and not to move, some artifacts appeared in each signal, mainly because of movements. This fact is not unique to our study; other researchers have also reported this issue [23, 24]. The usual method for detecting these parts in the signal is to compare all subsequent RR durations with a median duration, and if the absolute difference is higher than a threshold, then, that RR interval is dropped and it is skipped in the later computations. The condition for accepting an RR interval during our experiments was that MedRR/1.2 < RR < MedRR∗1.2, where MedRR is the median of all RRs (durations between subsequent peaks). This method is very similar to the artifact filtering techniques mentioned in other studies [24, 25].

### 2.2. Measuring PPG

To measure the PPG signal, we decided to use an iPhone 6 smartphone. The procedure was, as in other projects [12, 13, 26], that after switching on the flash, the light would go through a finger of the subject in question and with the camera nearby, the adsorption of the light could be measured.

The application was developed in the Swift programming language, which initialized the back camera input for the so-called “Slow-mo” capturing mode (240 Hz, 720 p). A callback method was called when a new image buffer was available with its timestamp, and with this callback method, the PPG signal was computed in real time. In our implementation, the CPU utilization was about 40–50%, while real-time PPG signal production, analysis, and some GUI feedback (signal plots) for the user were carried out.

From the images of the video signal, the luma component was examined (Y component of the supported 420YpCbCr8BiPlanarFullRange format). In other studies, similar luminosity or brightness data (or just the data of the red channel) were used for computations (in the RGB video recording mode) [27, 28]. It can be seen that these techniques are equivalent, because in the RGB mode, all the blue and green pixel values are zeros and, consequently, all the linear combinations of RGB channels will result in similar curves like those for luminosity. Another technically important fact is that all the automatic functions of the camera can be switched off programmatically (like auto-white balance and autoexposure). Here, the level of the flash (“torch”) was set to the maximum.

Unlike that for the ECG signal, here, not just data values but their corresponding timestamps were also available. So it may be interesting and important to investigate the spacing between the timestamps; namely, how much one differs from that of an equally spaced one. Because not just the durations between consecutive peaks (RR or NN intervals) but also their differences will be considered here, a large jump in the duration between timestamps could be a source of error. Fortunately, the durations between the consecutive timestamps have a very small variation. The maximum and minimum differences between consecutive time intervals are of the order of 1*e* − 7, which means that there is a fairly regular time spacing of the video stream signal.

The next preprocessing steps for the PPG signal were the same as those for ECG, namely, those of low-pass filtering, high-pass filtering, peak detection, and filtering out artifacts from the set of RR intervals.

### 2.3. Parallel Measurements

**Figure 3:**
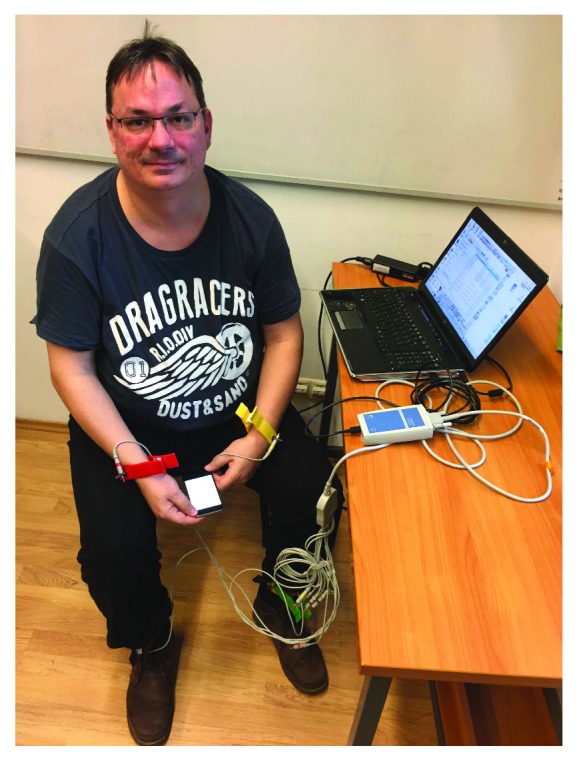
Experimental arrangement. The subject is sitting in a resting position, the electrodes of the ECG device are connected to the limbs, and the smartphone is held in the subject's palm.

**Figure 4:**
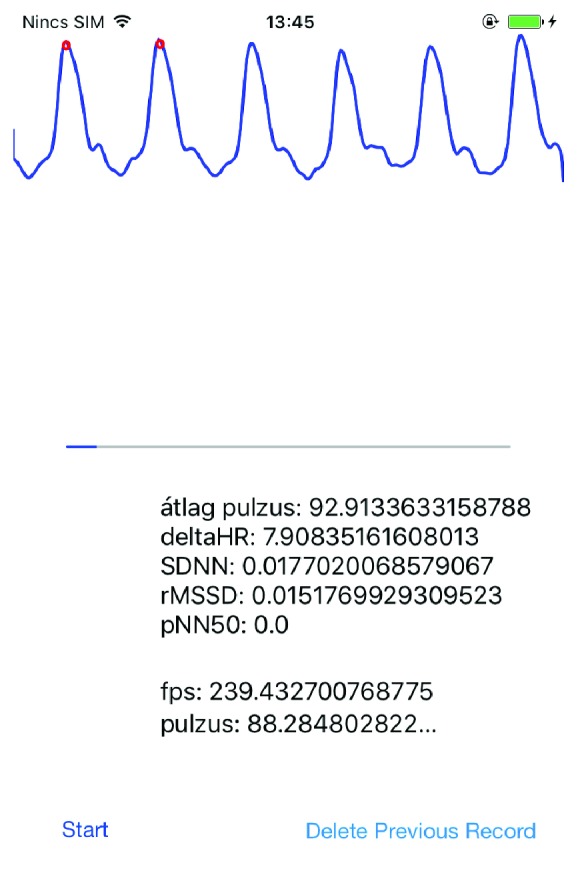
A screenshot of the PPG measurement application during a recording.

Many studies already confirm that HRV and PRV parameters, derived from the series of RR and PP durations, are consistent with each other [10, 12, 13]. Our aim here was to investigate this correspondence, when the PPG signal is obtained from the video stream with high frames per second using an iPhone 6. For the sake of a suitable comparison, parallel measurements were made using a standard ECG device and an iPhone 6 smartphone.  Figure 3 shows a typical scenario for this. The application developed for the iPhone was designed so that a measurement begins with a 20-second “practice” part, during which the subject can locate his/her finger on the back camera and the flash appropriately based on feedback (i.e., the filtered PPG signal is shown in real time on the GUI; see Figure 4 for a screenshot). Later, a tone is played, which indicates that the ECG measurement has also to be started. After 5 minutes, a second tone indicates that all the measurements have to be stopped (and the signals must be saved).

**Figure 5:**
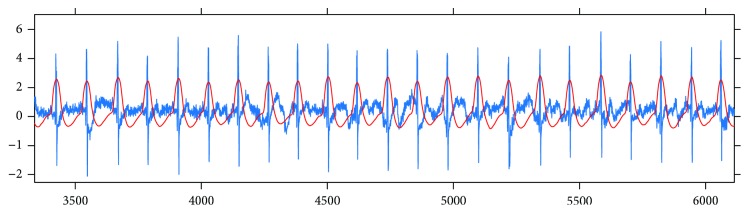
Illustration of the results of the synchronization process, with the ECG signal shown in blue and the PPG signal shown in red.

When evaluating the parallel measurements, both signals were processed using the methods described above; then, a parallel processing step was performed which attempted to remove from both RR and PP series those values that might correspond to artifacts in at least one of the ECG or PPG signals. For this, after the peaks were detected in both signals, a synchronization step was carried out. Namely, the peak series were paired to each other with a minimal error.  Figure 5 shows this synchronization step. The pairing process examined multiple parts taken from both signals to determine the optimal shift value between them, because the artifacts could be anywhere in a signal. Moreover, a time scaling multiplier was calculated, the value of which was very close to 1, since the sampling frequency of the ECG signal was not exactly 500 Hz and the FPS of the video stream was not exactly 240 Hz (actually, it was 239.84 Hz in our experiments).

After this pairing process, RR (and PP) durations corresponding to an artifact in one of the time series were removed. Another filter was applied that deleted RR and PP durations from both series, if they differed by more than 0.3 second.

### 2.4. Analysis of the Signals

**Figure 6:**
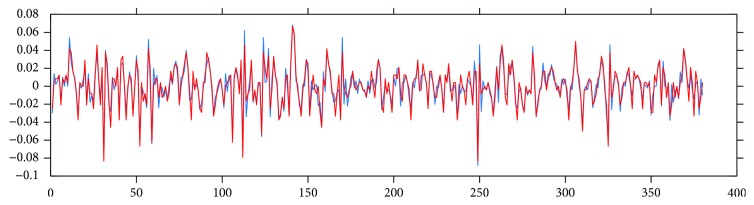
Delta RR duration series computed on ECG (blue) and PPG (red) signals.

There are many medically relevant parameters which can be derived from the RR series. Some of these parameters are statistical properties of the RR time series, while others characterize the frequency-domain features of the RR data. Some values measure statistical properties of the differences between consecutive RR durations.  Figure 6 shows this delta RR series computed on ECG and PPG signals.

When comparing the RR (PP) series got from ECG and PPG signals, the usual way is to compare the derived HRV (PRV) measures [1, 12]. Since one goal of this study was to compare our results with those of other ECG-PPG comparison studies, we computed the measures described in those studies. We collected the definitions of these parameters below (where *N* is the number of RR durations, RR*_i_* is the *i*th RR duration in ms, *P_i_* is the corresponding pulse value (60,000/RR*_i_*), and DRR*_i_* = RR_*i*+1_ − RR*_i_*). The abbreviations have the following meanings: standard deviation of RR interval time series (SDRR), root mean square of successive differences (RMSSD), and probability of the successive differences of NN (or RR) intervals which differ by more than 50 ms (pNN50).

(1) $$\begin{array}{c} \bar{RR}=\frac{1}{N}\overset{N}{\underset{i=1}{\sum }}{RR}_{i},\\ \bar{P}=\frac{1}{N}\overset{N}{\underset{i=1}{\sum }}p_{i},\\ SDRR=std\left({RR}_{i}\right),\\ RMSSD=\sqrt{\frac{1}{N-1}\overset{N}{\underset{i=1}{\sum }}{DRR}_{i}^{2}},\\ pNN50=P\left(\left|{DRR}_{i}\right|>50\;ms\right). \end{array}$$

The definitions of the frequency-domain parameters contain the *f*(*λ*) function, which is the power spectrum of the RR tachogram. The definitions of the abbreviations are the following. VLF stands for the power in the very low frequency range, LF represents the power in the low frequency range, and HF means the power in the high frequency range.

(2) $$\begin{array}{c} VLF=\int_{0.003\;Hz}^{0.04\;Hz}f\left(\lambda \right)d\lambda ,\\ LF=\int_{0.04\;Hz}^{0.15\;Hz}f\left(\lambda \right)d\lambda ,\\ HF=\int_{0.15\;Hz}^{0.4\;Hz}f\left(\lambda \right)d\lambda . \end{array}$$

In other studies, some of these parameters had a different name. For example, SDNN is the same as SDRR and the NN duration or the PP duration is equivalent to the RR duration. In different publications, AVNN (average of NN intervals) corresponds to the average RR (AVRR) or average PP (AVPP).

### 2.5. Comparison Methods

Two kinds of comparison methodologies are commonly used in the literature. The first is the Pearson correlation coefficient (given below) with linear regression parameters computed on the two series [29]:

(3) $$\begin{array}{c} PC=\frac{\sum_{i=1}^{n}\left(x_{i}-\bar{x}\right)\left(y_{i}-\bar{y}\right)}{\sqrt{\sum_{i=1}^{n}{\left(x_{i}-\bar{x}\right)}^{2}\sum_{i=1}^{n}{\left(y_{i}-\bar{y}\right)}^{2}}}. \end{array}$$

Because this correlation value was always close to the one in the experiments, but the differences of the PPG- and ECG-derived values displayed a clearly visible deviation, a more sophisticated plot and comparison method was introduced, called the Bland-Altman plot and analysis [30, 31]. The mathematical definitions of measurement values are the following:

(4) $$\begin{array}{c} Bias=\frac{1}{n}\overset{n}{\underset{i=1}{\sum }}\left(y_{i}-x_{i}\right),\\ SD=\sqrt{\frac{1}{n-1}\overset{n}{\underset{i=1}{\sum }}{\left(y_{i}-x_{i}-Bias\right)}^{2}},\\ LOA=Bias\pm 1.96\;SD,\\ AL=\pm \frac{1}{n}\overset{n}{\underset{i=1}{\sum }}\frac{y_{i}+x_{i}}{2},\\ BAR=\frac{1.96\;SD}{1/n\sum_{i=1}^{n}\left(y_{i}+x_{i}/2\right)}. \end{array}$$

Here, “bias” means an average shift in the values relating to the reference data (*x*), and SD denotes the standard deviation of the differences. Limit of agreement (LOA) stands for providing an agreement limit, when the distribution of differences is supposed to be a normal distribution. An acceptance limit (AL) is also introduced [12, 32], which is determined by the scale of the values of the reference and the ones examined (here, all the values are positive). The BAR (Bland-Altman ratio) parameter relates SD to AL, and it has been given a meaning [12, 33] that if the value is at most 10%, then, the agreement is ranked as good, and if the value is above, it is moderate (10% < BAR ≤ 20%) or insufficient (BAR > 20%).

Since both methods (correlation and Bland-Altman statistics) were used in different reports, we calculated all these statistical values for characterizing our measurements and for the sake of comparing our findings with those in the other studies.

## 3. Results and Discussion

**Figure 7:**
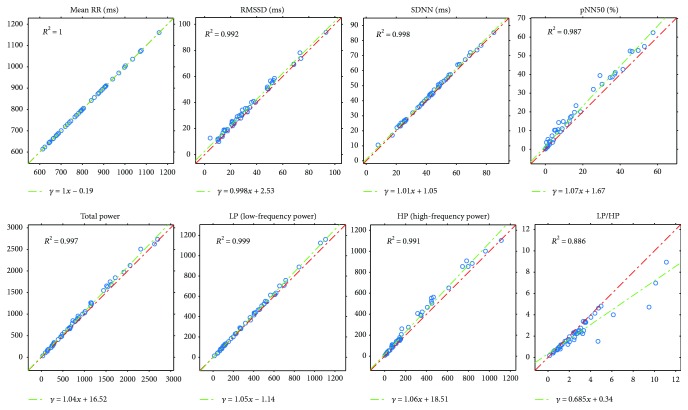
Plots of PRV indices related to HRV indices (horizontal axis) with *R*^2^ and linear regression.

**Figure 8:**
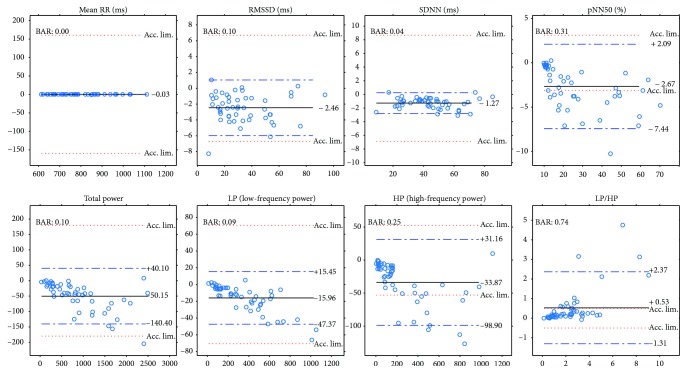
Bland-Altman plots for PRV and HRV indices with limits of agreement (blue dashed lines), bias (black lines), and acceptance limits (red dotted lines).

Next, we will present our results of all the computed comparison parameters defined above. These parameters will be computed not just for the PPG-ECG signal pairs but also for the ECG channel pairs. Moreover, figures will be included to show the linear relationship between the indices and the Bland-Altman plots.  Table 1 and the plots (Figures 7 and 8) show our HRV-PRV comparison results.

### 3.1. Results of Comparisons among ECG Channels

We mentioned previously that when comparing the parameters derived from PPG with those derived from ECG measurements, the ECG signal is treated to be a gold standard. However, a clinically used ECG device has more than one channel, and the question arises, of which channel should be used as the basis of a comparison process. Moreover, what if, when comparing the HRV indices corresponding to different ECG channels with each other, we have similar properties, like when we compare HRV with PRV?

**Figure 9:**
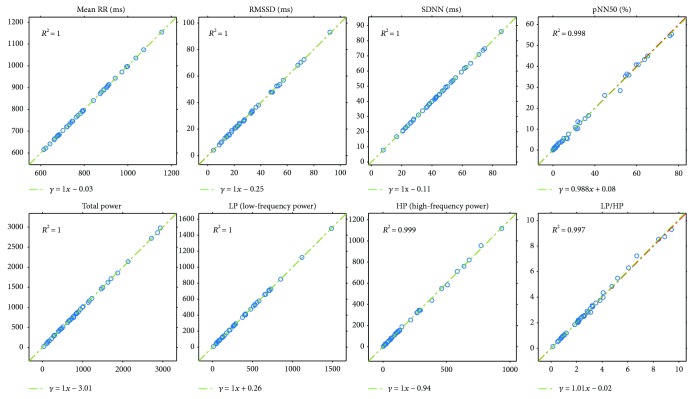
Plots of HRV indices calculated for ECG channel 1 and ECG channel 2 with *R*^2^ and linear regression.

**Figure 10:**
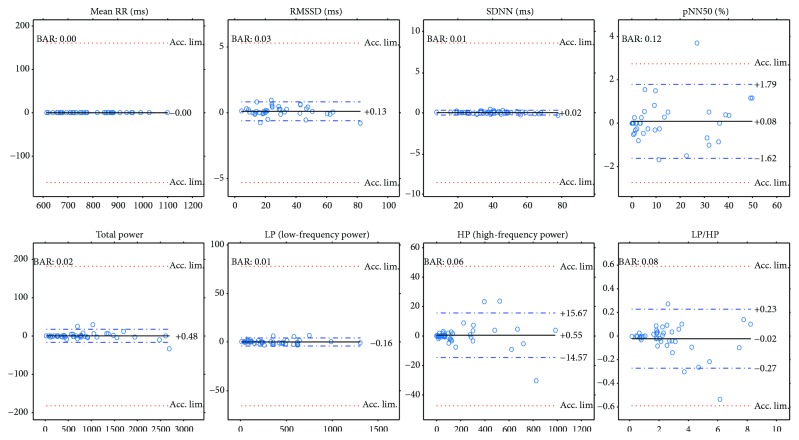
Bland-Altman plots of HRV indices calculated for ECG channel 1 and ECG channel 2 with limits of agreement (blue dashed lines), bias (black lines), and acceptance limits (red dotted lines).

**Figure 11:**
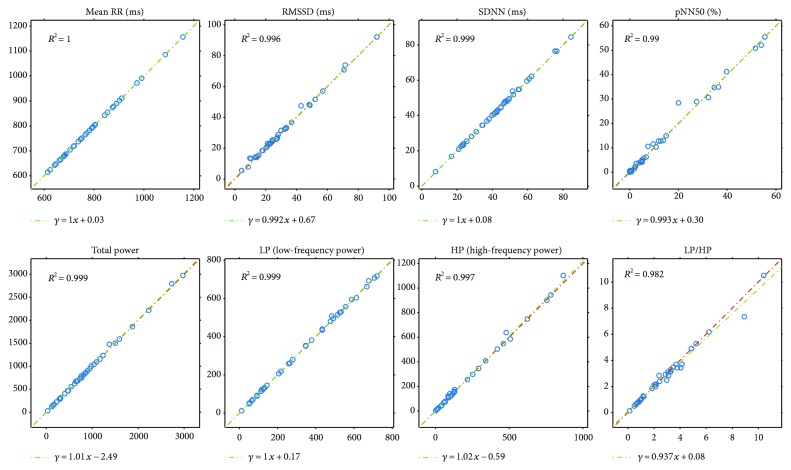
Plots of HRV indices calculated for ECG channel 1 and ECG channel 3 with *R*^2^ and linear regression.

**Figure 12:**
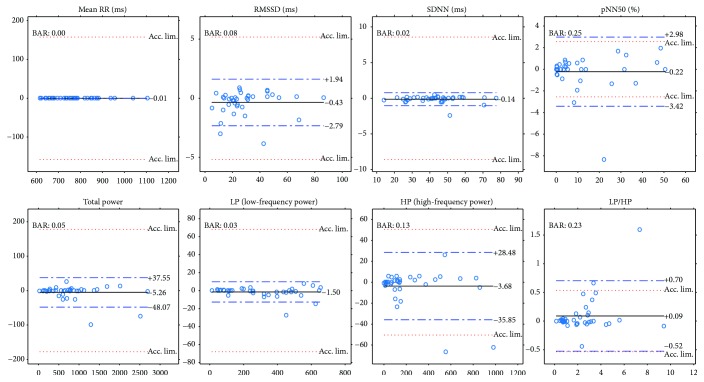
Bland-Altman plots of HRV indices calculated for ECG channel 1 and ECG channel 3 with limits of agreement (blue dashed lines), bias (black lines), and acceptance limits (red dotted lines).

**Figure 13:**
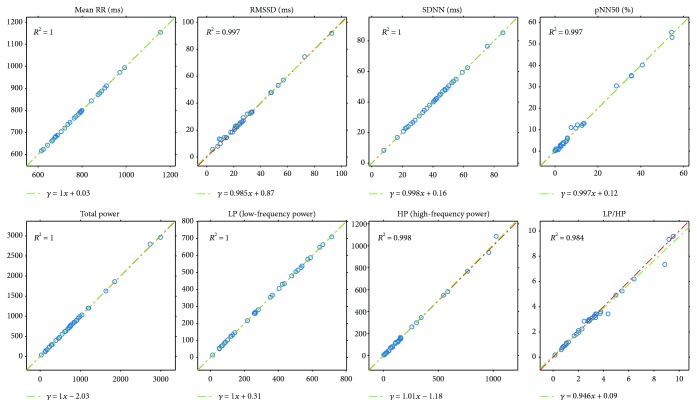
Plots of HRV indices calculated for ECG channel 2 and ECG channel 3 with *R*^2^ and linear regression.

**Figure 14:**
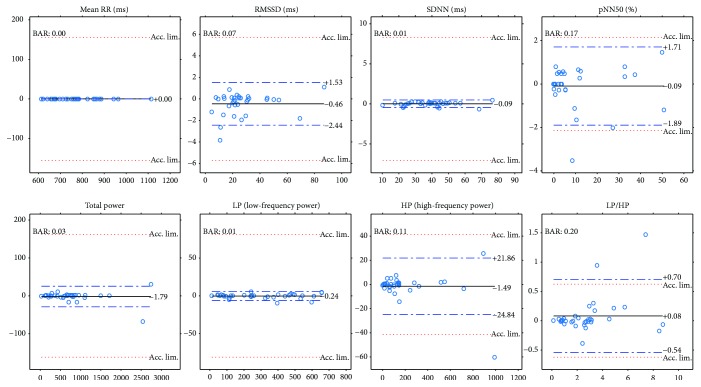
Bland-Altman plots of HRV indices calculated for ECG channel 2 and ECG channel 3 with limits of agreement (blue dashed lines), bias (black lines), and acceptance limits (red dotted lines).

In the experiments, a Cardiax PC-ECG device was used that had four electrodes connected to the four limbs of the participants. This resulted in three channel data. Figures 9 and 10 show the same plots for the ECG(1)-ECG(2), as those for ECG-PPG (Figures 7 and 8). Figures 11 and 12 and Figures 13 and 14 show these results for the ECG(1)-ECG(3) and ECG(2)-ECG(3), respectively. Some key values are highlighted in the figures, and the relevant ones are listed in Table 2 in Discussion.

## 4. Discussion

Next, we will examine other studies to determine the position of our results relative to these. Furthermore, we will discuss the point that the ECG channels differ from each other, and this means that in an ECG-PPG comparison, this should be taken into account.

### 4.1. Our Result in Itself

Our results reveal a good correspondence between most indices of HRV and PRV (see Table 1 and Figures 7 and 8). Most of the correlations are above 0.99, and ln(RMSSD) and TP/HP have slightly lower correlation values. What is more, the Bland-Altman analysis also provides good results. The agreement is insufficient (BAR > 20%) just for pNN50, HP, and HP/LP parameters. The reason for this is the high bias, which is probably due to the influence of breathing on the high-frequency PRV components.

### 4.2. Comparison with Smartphone-Based PRV-HRV Correspondence Measurements

The authors of various studies have reported comparison results between the analyses of ECG and PPG signals. Among these studies, there are a few reports that describe measurements of the PPG signal using a smartphone. In a study [12], the authors used an HTC S510e smartphone to take PPG measurements (20–30 FPS) and a Finometer MIDI as an ECG data acquisition tool (200 Hz). The number of participants was 30, and the duration of the recordings was at least 5 minutes. They found a perfect correlation for just the AVNN time-domain parameter, but other correlations between time-domain indices were 0.933, 0.78, and 0.5 for the SDNN, RMSSD, and PNN50 indices, respectively. Our results for these correlations are 0.999, 0.996, and 0.993, respectively, which are much better results. The linear regression parameters display a much greater difference between the indices than those in our findings, which are summarized in Table 1. Surprisingly in the frequency domain, their HRV and PRV indices correlate better, but in the case of 5-minute measurements, the VLF power (power in the very low frequency range, 0.003–0.04 Hz) computation is not very useful (the authors gave this value in their study). The Bland-Altman analysis revealed similar findings in the time domain (they got worse results than ours) and in the frequency domain, as well. For example, their BAR value for SDNN is 19.17%; for RMSSD, 42.22%; and for PNN50, 79.91%, while our corresponding values are 3.5%, 10.6%, and 30.6%, respectively.

Another study [13] reported an experiment using iPhone 6 for PPG and a 12-lead ECG treadmill (GE Series 2000, GE Medical Systems Information Technologies Inc., Milwaukee, WI, USA) for HR measurements. They compared just the accuracy of heart rate estimates got from the two kinds of signals. In a resting position situation, they found a 0.993 correlation with a mean difference of −0.05 beats/min and a standard deviation of 1.03 beats/min. Our corresponding values for these parameters are 1 for the correlation, 0.032 beats/min for the bias, and 0.11 beats/min for the standard deviation.

In a third experiment [21], 30 participants were involved in measuring their ECG and PPG in parallel, using a Biopac ECG and an iPad2 combined with an infrared pulse sensor (ithlete™). They compared indices computed from ultra-short-term signals (of approximately one minute in length). They got a bias of 0.94 and a standard deviation of 1.77 on the ln(RMSSD) index differences (when the measurements were taken in a seated position). These values are higher than ours (0.103, 0.153), which indicate a significantly worse results.

In Table 2, we collected all the data that could be accessed in previous publications on the topic of comparing smart device-based PPG measurements with ECG. The better values are shown in bold.

In Table 2, the results are an order of magnitude better when our measurements are compared to those in [13] or in [21]. Our results are significantly better compared to those of some important parameters examined in [12], but our high-frequency-domain parameters (HP, LP/HP) are much worse. We do not know the precise reason for this; perhaps, the authors of [12] described a special regulated breathing for the subjects of the experiments. The kind of breathing (spontaneous or regulated) during the experiments can influence the high-frequency-domain power spectrum.

### 4.3. Our Results in Relation to ECG-ECG Correspondence

Another topic in this study was not just to compare the parameters computed from a PPG signal with those computed from an ECG channel but also to investigate those values related to an ECG-to-ECG channel comparison. The results given in the previous sections (Figures 9–14) tell us that the HRV parameters (or indices) computed via an analysis of an ECG channel differ from each other for different channels. In our experiments here, the ECG(1)-ECG(2) channel comparison had the lowest standard deviation values on Bland-Altman difference plots, and the ECG(1)-ECG(3) differences were the highest. In Table 3, we list the correspondences for the most important indices for the ECG-PPG and the three ECG-ECG comparisons.

The results indicate a good agreement for the parameters mean RR, SDNN, TP, and LP. There is a moderate agreement for RMSSD in the PRV-HRV comparison, but the BAR value is not much higher than that for the ECG(1)-ECG(3) comparison. The agreements are insufficient for the PNN50 and HP values (PPG-ECG), but these are also insufficient in the ECG comparisons. In the PPG-ECG comparison, a significant bias was found for some HRV indices, which are not given in ECG(i)-ECG(j) comparisons. This means that PNN50 and the spectral parameters (TP, LP, and HP) are overestimated, especially when the reference values are large. This phenomenon is clearly visible in Figure 8. Other studies also mention this fact (for references, see [9]). In the latter study, the authors offer an explanation for this observation: “The fact that spontaneous breathing rates usually lie within the HF frequency band explains why many studies found that PRV overestimates HRV mostly in the HF domain or in variables reflecting short-term variability (HF, RMSSD, pNN50, etc.).”

In order to summarize the most important analysis values of the Bland-Altman method, we collected the SD (standard deviation) and BAR (Bland-Altman ratio) values for the various HRV indices corresponding to the ECG(i)-ECG(j) and ECG-PPG comparisons. From the ECG(i)-ECG(j) values, the worst were taken (which are in bold in Table 3). We also computed the ratio of the ECG-ECG and ECG-PPG values.  Table 4 contains data concerning this comparison.

Earlier, we found that there is a significant bias between some ECG- and PPG-based variability indices.  Table 4 tells us that for the time-domain indices, the standard deviation of the differences (SD) and the Bland-Altman ratio (BAR) corresponding to PPG indices are at most two times higher than those corresponding to ECG. This factor is slightly above two for the frequency-domain indices. We think that this correspondence between the HRV and PRV should suffice for an application if we wish to collect PRV data from a larger group worldwide.

## 5. Conclusions

In order to achieve our main goal, one of the first steps was to compare PRV with HRV. Our results indicate that almost all the indices computed from PRV may be an alternative to those computed from HRV, even for clinical use. This may be concluded from the results of our comparison among the PRV-HRV correspondences and HRV-HRV correspondences. However, there are some indices which show a bias related to the values computed from an HRV analysis (mainly pNN50 and high-frequency power). This phenomenon corresponding to biases was found in other earlier studies as well [9], so one might think that with some direct (possibly) linear transformation or by taking into account the rhythm of breathing, there should be a way to minimize the errors between the two kinds of rate variability indices. In the future, we plan to validate PRV measured using a smartphone with HRV involving CAD (coronary artery disease) patients. Moreover, we are interested in whether there is any medical reason which explains the variability among the derived indices computed from different ECG channels. Also a short-term goal of ours is to make our smartphone application free to the public and get as many people involved in data collection as possible.

## Figures and Tables

**Table 1:** Comparison values when PRV and HRV indices are compared with each other. Here, PC stands for the Pearson correlation coefficient (with the *P* values), *m* and *b* represent the coefficients for the linear regression on HRV (PRV) with the corresponding mean error (err), *R*^2^ is the coefficient of determination, and bias, SD, and BAR values are the results of the Bland-Altman analysis. The definitions of the HRV indices were introduced earlier.

	PC	*P*	lin. *m*	lin. *b*	lin. err (MSE)	lin. *R*^2^	Bias	SD	BAR
HR (beat/min)	1	<10^−23^	1.00	−0.12	0.011	1	0.032	0.110	<0.001
Mean RR(ms)	1	<10^−23^	1.00	−0.02	0	1	−0.002	0.009	<0.001
RMSSD (ms)	0.996	<10^−23^	1.00	2.53	3.15	0.992	2.464	1.793	0.106
ln(RMSSD)	0.973	<10^−23^	0.87	0.528	0.017	0.947	0.103	0.153	0.089
SDNN (ms)	0.999	<10^−23^	1.01	1.06	0.582	0.998	1.271	0.776	0.035
pNN50 (%)	0.993	<10^−23^	1.07	1.67	4.399	0.987	2.673	2.432	0.306
TP (total power, ms^2^)	0.998	<10^−23^	1.04	16.52	1439.3	0.997	50.15	46.05	0.100
LP (lf power, ms^2^)	0.999	<10^−23^	1.04	−1.14	86.63	0.999	15.96	16.03	0.089
HP (hf power, ms^2^)	0.995	<10^−23^	1.06	18.51	783.9	0.991	33.87	33.18	0.246
LP + HP	0.998	<10^−23^	1.06	13.14	1124.4	0.996	49.83	45.21	0.144
LP/HP	0.941	<10^−23^	0.68	0.341	0.326	0.885	−0.529	0.937	0.736

**Table 2:** HRV and PRV index comparisons can be found in the corresponding literature (in parentheses). PC stands for the Pearson correlation coefficient (with the *P* values), and bias, SD, and BAR values are the results of the Bland-Altman analysis. The definitions of the HRV indices were introduced by us earlier.

Derived index comparison	Cited value	Our value
SDNN-PC ([12])	0.933	**0.999**
SDNN-BAR ([12])	19.17%	**3.5**%
RMSSD-PC ([12])	0.78	**0.996**
RMSSD-BAR ([12])	42.22%	**10.6**%
pNN50-PC ([12])	0.5	**0.993**
pNN50-BAR ([12])	79.91%	**30.6**%
LP-PC ([12])	0.996	**0.999**
LP-BAR ([12])	12.14%	**8.9**%
HP-PC ([12])	**0.996**	0.995
HP-BAR ([12])	**10.22**%	25.6%
LP/HP-PC ([12])	**0.982**	0.941
LP/HP-BAR ([12])	**19.3**%	73.6%
avg(PP)-PC ([13])	0.993	**1**
avg(PP)-bias ([13])	−0.05 beats/min	**0.032** beats/min
avg(PP)-SD ([13])	1.03 beats/min	**0.11** beats/min
ln(RMSSD)-bias ([21])	0.94	**0.103**
ln(RMSSD)-SD ([21])	1.77	**0.153**

**Table 3:** Comparison values for the correspondence values among PRV and HRV indices, when comparing PPG to ECG, and the channels of ECG. Here, PC stands for the Pearson correlation coefficient (with *P* values smaller than 10^−10^), *m* and *b* represent the coefficients for the linear regression, and bias, SD, and BAR values are for the Bland-Altman analysis. The worst values are in bold.

		ECG-PPG	ECG(1)-ECG(2)	ECG(1)-ECG(3)	ECG(2)-ECG(3)
Mean RR (ms)	PC	1	1	1	1
	*m*	1	1	1	1
	*b*	−0.019	−0.025	0.028	**0.032**
	Bias	−0.021	0.0015	**0.007**	−0.003
	SD	0.0093	0.021	**0.061**	0.026
	BAR	<0.001	<0.001	<0.001	<0.001

RMSSD (ms)	PC	0.996	1	**0.998**	0.999
	*m*	0.998	1.004	0.99	0.99
	*b*	2.53	−0.25	**0.67**	0.87
	Bias	2.64	−0.13	0.43	**0.46**
	SD	1.79	0.44	**1.21**	1.01
	BAR	0.104	0.027	**0.076**	0.07

SDNN (ms)	PC	0.998	1	1	1
	*m*	1.005	**1.002**	1.001	0.998
	*b*	1.055	−0.113	0.075	**0.162**
	Bias	1.27	−0.020	**0.136**	0.092
	SD	0.776	0.148	**0.464**	0.260
	BAR	0.035	0.0068	**0.021**	0.012

pNN50 (%)	PC	0.993	0.999	**0.995**	0.998
	*m*	1.07	0.988	**0.993**	0.997
	*b*	1.67	0.076	**0.305**	0.122
	Bias	2.67	−0.083	**0.221**	0.094
	SD	2.43	0.869	**1.631**	0.918
	BAR	0.306	0.125	**0.250**	0.168

TP (total power, ms^2^)	PC	0.998	1	1	1
	*m*	1.04	1.003	**1.009**	1.005
	*b*	16.52	−**3.01**	−2.49	−2.032
	Bias	50.15	−0.484	**5.261**	1.788
	SD	46.15	8.768	**21.84**	13.83
	BAR	0.10	0.019	**0.048**	0.0335

LP (low-frequency power, ms^2^)	PC	1	1	1	1
	*m*	1.05	1	**1.004**	1
	*b*	−1.14	0.26	0.167	**0.307**
	Bias	15.96	0.16	**1.50**	0.243
	SD	16.03	2.26	**5.74**	2.43
	BAR	0.089	0.012	**0.033**	0.015

HP (high-frequency power, ms^2^)	PC	0.995	1	0.999	0.999
	*m*	1.06	1.002	**1.017**	1.013
	*b*	18.514	−0.94	−0.586	−**1.18**
	Bias	33.87	−0.55	**3.68**	1.49
	SD	33.18	7.71	**16.41**	11.91
	BAR	0.247	0.064	**0.13**	0.11

**Table 4:** Bland-Altman SD and BAR values for the ECG-PPG and the worst ECG(i)-ECG(j) correspondences.

		ECG-PPG	Worst ECG(i)-ECG(j)	(1)/(2)
Mean RR (ms)	SD BAR	0.0093 <0.001	0.061 <0.001	0.15 n/a
RMSSD (ms)	SD BAR	1.79 0.104	1.21 0.076	1.48 1.37
SDNN (ms)	SD BAR	0.776 0.035	0.464 0.021	1.67 1.67
pNN50 (%)	SD BAR	2.43 0.306	1.631 0.250	1.49 1.22
TP (total power, ms^2^)	SD BAR	46.15 0.10	21.84 0.048	2.11 2.08
LP (low-frequency power, ms^2^)	SD BAR	16.03 0.089	5.74 0.033	2.79 2.70
HP (high-frequency power, ms^2^)	SD BAR	33.18 0.247	16.41 0.13	2.02 1.9
